# AAV mediated repression of Neat1 lncRNA combined with F8 gene augmentation mitigates pathological mediators of joint disease in haemophilia

**DOI:** 10.1111/jcmm.18460

**Published:** 2024-06-12

**Authors:** Pratiksha Sarangi, Mohankumar B. Senthilkumar, Sonal Amit, Narendra Kumar, Giridhara R. Jayandharan

**Affiliations:** ^1^ Laurus Center for Gene Therapy, Department of Biological Sciences and Bioengineering and Mehta Family Centre for Engineering in Medicine and Gangwal School of Medical Sciences and Technology Indian Institute of Technology Kanpur Kanpur Uttar Pradesh India; ^2^ Department of Pathology Autonomous State Medical College Kanpur Uttar Pradesh India

**Keywords:** adeno‐associated virus, F8, haemophilic arthropathy, matrix metalloproteinases, murine model, *Neat1*, shRNA

## Abstract

Haemophilic arthropathy (HA), a common comorbidity in haemophilic patients leads to joint pain, deformity and reduced quality of life. We have recently demonstrated that a long non‐coding RNA, *Neat1* as a primary regulator of matrix metalloproteinase (MMP) 3 and MMP13 activity, and its induction in the target joint has a deteriorating effect on articular cartilage. In the present study, we administered an Adeno‐associated virus (AAV) 5 vector carrying an short hairpin (sh)RNA to *Neat1* via intra‐articular injection alone or in conjunction with systemic administration of a capsid‐modified AAV8 (K31Q) vector carrying F8 gene (F8‐BDD‐V3) to study its impact on HA. AAV8K31Q‐F8 vector administration at low dose, led to an increase in FVIII activity (16%–28%) in treated mice. We further observed a significant knockdown of *Neat1* (~40 fold vs. untreated injured joint, *p* = 0.005) in joint tissue of treated mice and a downregulation of chondrodegenerative enzymes, MMP3, MMP13 and the inflammatory mediator‐ cPLA2, in mice receiving combination therapy. These data demonstrate that AAV mediated *Neat1* knockdown in combination with F8 gene augmentation can potentially impact mediators of haemophilic joint disease.

## Graphical abstract

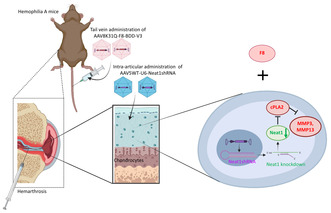

## 1 INTRODUCTION

Haemophilia A and B affects 1 in 5000 and 1 in 20,000 males all over the world, respectively.^1^ It is caused due to mutations in the factor (F) 8 or F9 gene(s), leading to a deficiency of clotting factor VIII or IX, respectively.^2^ Patients with severe haemophilia (<1% of coagulant activity) have repeated episodes of acute, spontaneous bleeding into the joint cavity, ranging between 50 and 70 bleeds per year.^3^, ^4^ Recurrent bleeding into the same joint over time, causes the joint to deteriorate and eventually results in haemophilic arthropathy (HA).^5^ Previous studies in murine models of HA found evidence of the involvement of the nuclear factor kappa light chain enhancer of activated B cells (NF‐kB) signalling pathway in the development of this debilitating condition. Hemosiderin deposits from RBCs along with infiltrated macrophages in the synovium activate NF‐kB signalling through inflammatory cytokines.^6^ We have demonstrated that overexpression of miR125a‐5p in tandem with F8 gene transfer using AAV vector diminished joint damage progression by downregulating the molecular mediators involved in the disease pathogenesis including MMP3, 9 and 13.^7^ In our previous study, we have also highlighted the emerging role of long non‐coding RNAs (lncRNAs) *H19* and *Neat1* in a mouse model of chronic HA.^8^ The function of similar lncRNAs in other joint diseases such as rheumatoid arthritis (RA) and osteoarthritis (OA), in terms of disease onset and advancement, has been extensively studied.^9^, ^10^, ^11^, ^12^, ^13^ The dysregulation of lncRNAs, including *Neat1*, *H19*, *Mir22hg* and *Malat1*, in collagen‐induced arthritis (CIA), OA and RA patients or in animal models, has also been reported.^12^, ^14^, ^15^, ^16^

*Neat1* (nuclear enriched abundant transcript 1) lncRNA has a crucial role in maintaining the integrity of the nuclear paraspeckle substructure.^17^ *Neat1* is known to be upregulated (~1.5 fold) in joint tissues of both RA and OA patients.^10^, ^13^, ^15^ *Neat1* has a significant impact on inflammation, chondrocyte apoptosis, and the degradation of cartilage.^13^, ^15^ *Neat1* lncRNA is known to alter the expression of cytosolic phospholipase A2 (cPLA2), an enzyme encoded by *Pla2g4a* by sequestering miR‐543.^13^ Recent research also suggests that lncRNAs may have therapeutic potential for joint diseases like OA and RA.^18^, ^19^, ^20^ For example, *Neat1* lncRNA has been implicated in the pathogenesis of OA and has been postulated as a potential therapeutic target.^20^ Whilst these studies have provided valuable insights into the crucial role of *Neat1* lncRNA in other joint diseases, the role of targeting *Neat1* lncRNA in the context of HA remains to be explored. In the present study, we studied the impact of combination gene therapy in haemophilia A mice, by Adeno associated virus (AAV) mediated knockdown of *Neat1* lncRNA within the articular cartilage, and liver directed F8 gene therapy in HA.

## 2 MATERIALS AND METHODS

### 2.1 Design and development of *Neat1* specific short hairpin (sh)RNA vector

The shRNA sequence (48 bp) for targeting mouse *Neat1* was obtained from *Wang* et al.,^21^ and cloned into an AAV backbone containing U6 promoter as illustrated in Figure S1A. The presence of the insert was validated by restriction digestion and Sanger sequencing. The shRNA had previously been validated to target mouse *Neat1* using AAV vector, so no scrambled shRNA was used as a control in this study.^21^ The expression of Neat1 shRNA was confirmed by transfection of the plasmid construct in murine fibroblast cells (NIH3T3) using Fugene^HD^ (Promega, Wisconsin, USA). After 48 h of transfection, RNA was isolated using Trizol (Invitrogen, Massachusetts, USA) and cDNA was prepared from 1 μg of RNA using Quantitect reverse transcription kit (Qiagen, Hilden, Germany). The levels of *Neat1* was measured by quantitative (q)PCR using commercially available mouse *Neat1* specific primers (Qiagen, Geneglobe ID‐SBM0868233). *Gapdh* (Qiagen, Geneglobe ID‐ SBM1220562) was used as reference gene for normalisation of qPCR data.

### 2.2 Vector production

The Neat1 shRNA and the F8‐BDD‐V3 constructs were packaged into AAV5WT and AAV8 mutant capsid (AAV8K31Q), respectively as previously described.^22^, ^23^ AAV vectors were produced by triple transfection of capsid, transgene, and helper plasmid in a 1:1:1 ratio in a producer cell line (AAV293) using polyethyleneimine (Polysciences Inc, Pennsylvania, USA). After 72 h of transfection, cells were harvested and lysed by freeze–thaw cycles in liquid nitrogen/ dry‐ice ethanol bath. The cell lysate was subjected to benzonase digestion (Santacruz Biotechnology, Santacruz, California, USA) followed by iodixanol gradient ultracentrifugation. The virus was then purified by column chromatography (Cytiva, Massachusetts, USA) and concentrated using Amicon filter (Merck Millipore, Massachusetts, USA). The viral titres were quantified by qPCR using ITR specific primers with AAV2‐RSS (ATCC, Virginia, USA) as a standard.

### 2.3 Repression of Neat1 lncRNA in combination with factor 8 (F8) gene augmentation in mice model of HA

**FIGURE 1:**
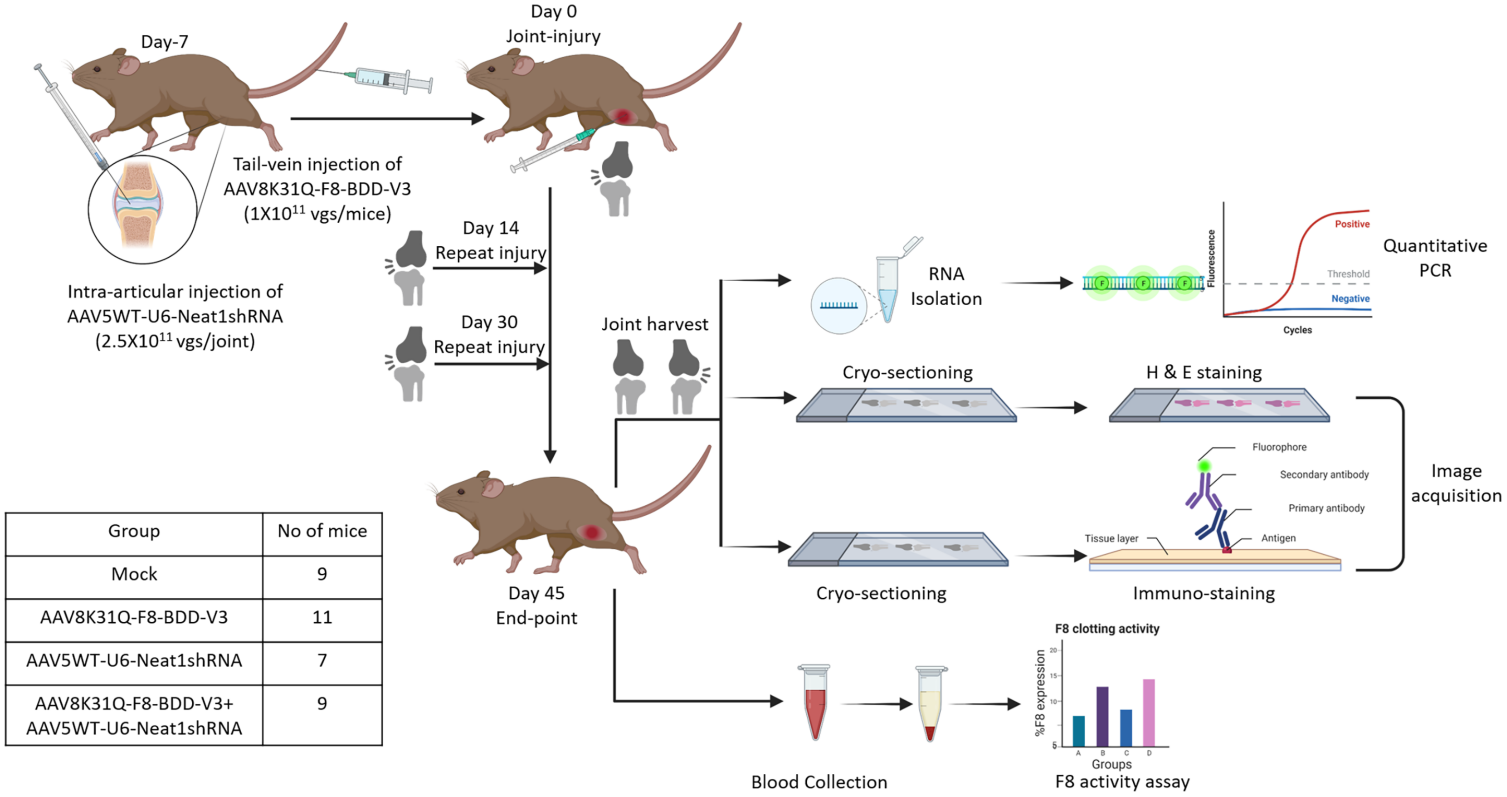
Study design to evaluate the impact of AAV based inhibition of lncRNA Neat1 in combination with F8 gene augmentation in a murine model of haemophilic arthropathy. Haemophilia A mice were divided into 4 groups: Mock (*n* = 9), F8 (*n* = 11) treated, Neat1shRNA treated (*n* = 7), and F8 and Neat1shRNA treated (*n* = 9). AAV5WT‐U6‐Neat1shRNA was administered intra‐articularly in the right knee at a dose of 2.5 × 10^11^ vgs/joint and AAV8K31Q‐F8‐BDD‐V3 was injected systemically via tail vein at a dose of 1 × 10^11^ vgs/mouse. Mock treated mice received PBS injection. After 7 days of vector administration (Day 0), joint injury was performed in the right knee whereas the left knee was uninjured and served as control. Repeated joint bleeding episodes were induced on Day 14 and Day 30 to mimic chronic hemarthropathy like condition. On Day 45, blood was collected to measure FVIII activity level and joint tissues were harvested for assessment of *Neat1* expression. Immunohistochemical analysis was performed to check the expression of target proteins in the articular cartilage and histopathological assessment was done to examine the extent of joint damage. Image created using Biorender.com.

Haemophilia A mice (B6;129S‐F8^tm1Kaz^/J) and C57BL/6J mice (Jackson Laboratory, Maine, USA) were housed in animal facility with food and water provided ad libitum. The animal experiments were approved by Institutional Animal Ethics Committee, IIT Kanpur. Haemophilia A mice were administered with AAV8K31Q‐F8‐BDD‐V3 at a low dose of 1 × 10^11^ vgs/mouse and AAV5WT‐U6‐Neat1 shRNA at a dose of 2.5 × 10^11^ vgs/joint. Groups of animals received PBS (Mock group, *n* = 9), or AAV8K31Q‐F8‐BDD‐V3 by tail vein injection (F8 treated group, *n* = 11), or AAV5WT‐U6‐Neat1 shRNA (Neat1 shRNA group, *n* = 7) by intra‐articular administration of 10 μL volume of the vector into their right knee joints, or the combination of AAV8K31Q‐F8‐BDD‐V3 and AAV5WT‐U6‐Neat1 shRNA vectors via systemic injection and intra‐articular injection in right knee joint, respectively (*n* = 9). We then performed joint injuries to recapitulate HA in the murine model of haemophilia A as described earlier.^24^ Briefly, a week after vector administration, the first joint injury (Day0) was performed in the right knee of the mice using a 31G needle, whereas the contralateral left joint that was uninjured served as the control.^25^ The joint injury was repeated on Day14 and Day30 to recapitulate the pathophysiology of multiple bleeding episodes. Blood samples were collected, 52 days post‐vector administration for FVIII specific clotting assays. Joint tissue from the experimental animals was harvested for RNA isolation, immunohistochemistry and histopathology (Figure 1).

### 2.4 RNA isolation

The vector administered injured (right knee) and uninjured joints (contralateral left knee) (*n* = 4 mice) from each group were pooled for RNA extraction, respectively. The joint capsule was isolated by a resection of the femur distal end and below the tibia proximal end. This tissue was then immediately transferred to RNA protect reagent (Qiagen). The surrounding tissue consisting of muscle and synovium was excised and cartilage tissue was extracted from femoral condyles and tibial plateau. Total RNA was isolated using Trizol reagent (Invitrogen, Massachusetts, USA), as per manufacturer's protocol. cDNA was synthesized using Quantitect reverse transcription kit (Qiagen) for quantification of *Neat1* across different groups of mice.

### 2.5 Factor VIII assays

Blood samples from experimental mice were collected in 3.2% sodium citrate and plasma was isolated for the clotting assay. FVIII specific clotting activity was measured using the STA‐Deficient FVIII plasma (Diagnostica Stago, Asnières‐sur‐Seine, France) as per the manufacturer's protocol.^26^

### 2.6 Histological assessment

The control and injured joint tissues were harvested after 52 days of vector administration followed by multiple bleeding episodes. The joints were fixed in 4% paraformaldehyde (PFA) for 48 h, then decalcified in 14% ethylene diamine tetra acetic acid (EDTA) for 10–14 days. Further, decalcified tissues were dehydrated in sucrose gradient and cryosections (Leica CM1520, Leica Biosystems, Wetzlar, Germany) of ~10 μm thickness were obtained using polyfreeze (Sigma Aldrich, Missouri, USA). The joint sections were stained by haematoxylin and eosin staining and analysed for joint characteristics, as described previously.^27^ We also performed a Prussian blue staining as per standard protocol^28^ to document the iron deposits in the joint space.

### 2.7 Immunohistochemistry

Joints were collected from experimental mice that had three bleeding episodes (Day0, Day14 and Day30) and at Day45 after the first injury. The harvested joint tissues were processed and fixed in 4% paraformaldehyde for 48 h at 4°C. Further, decalcification of the joint tissues was carried out in 14% EDTA for 10–14 days. The joint tissues were then dehydrated in sucrose gradient overnight and blocks were prepared using polyfreeze (Sigma Aldrich). Cryosectioning was performed to obtain joint sections of ~10 μm thickness. Prior to staining, sections were fixed in 4% PFA and washed in phosphate buffered saline containing 0.1% Tween 20 (1X PBST). For immunostaining, sections were incubated with primary antibodies to cPLA2 (1:100, sc‐454) (Santacruz Biotechnology), MMP3 (1:50, sc‐21,732) and MMP13 (1:100, ab39012) (Abcam, Cambridge, United Kingdom) for 24 h at 4°C. Next, tissue sections were washed in 1X PBST and incubated with blocking buffer containing normal goat serum (Abcam) overnight at 4°C. Further, secondary antibody [Alexafluor 568 (1:500, A‐11004) (Invitrogen) or goat anti‐rabbit Cy3 (1:200, 111–165‐008) (Jackson Immuno Research, West Grove, Pennsylvania, USA)] was added to the tissue sections and incubated for 1 h at room temperature. This was followed by staining with 4′,6‐diamidino‐2‐phenylindole (DAPI) (Sigma Aldrich) and mounting was performed using Fluorsave (Merck Millipore). Fluorescent images were acquired using a confocal imaging system (LSM780NLO, Carl Zeiss GmbH, Wein, Austria) for visualisation of protein expression. For quantification of Alexafluor 568 or Cy3 signal, the fluorescence intensity was measured using ImageJ software as described previously. Briefly, a region of interest (ROI) was defined within the articular cartilage from the surface to the tide mark for all fluorescence images. The integrated density of Alexafluor 568 or Cy3 within the ROI was determined and used for statistical analysis. The exposure settings were kept uniform among various groups.^29^

### 2.8 Data analysis

Data are represented as mean ± SD. GraphPad Prism 8.0.2 was used to create all of the graphs, and Biorad CFX Manager 3.1 was used to analyse the gene expression data from quantitative PCR. An unpaired Student's *t*‐test was performed for statistical analysis. *p* ≤ 0.05 was considered as statistically significant.

## 3 RESULTS

### 3.1 Design of shRNA vector targeted against *Neat1* and its validation in vitro

Our earlier data had demonstrated that *Neat1* was substantially over expressed in the hemarthritic joints in a murine model of haemophilia A.^8^ To investigate if downregulation of *Neat1* is beneficial, we first cloned a previously reported shRNA sequence (48 bp) targeting mouse *Neat1* ^21^ in an Adeno‐associated virus (AAV) backbone containing a ubiquitous (U6) promoter (241 bp) (Figure S1A). The shRNA against *Neat1* used in this study was previously validated to be specifically targeting mouse *Neat1* and hence, we did not use a scrambled shRNA as control for validation study.^21^ To further validate this construct we confirmed the expression of the target sequence and its effect on *Neat1* by transient transfection of the cloned shRNA vector into murine fibroblast (NIH3T3) cells (Figure S1A). After 48 h, the relative levels of *Neat1* was ~1.95 fold (*p* = 0.0001) lower in shRNA transfected cells, when compared to mock transfected cells (Figure S1B). Similar level of *Neat1* knockdown by transient transfection of shRNA has been demonstrated in mouse cell lines in earlier studies.^30^, ^31^ The level of shRNA mediated knockdown of *Neat1* depends on the transfection efficiency and permissivity of cell line.^32^, ^33^ However, we observed a significant level of knockdown in vitro consistently (*n* = 3 biological replicates). This is comparable to the data obtained earlier with this target shRNA sequence in mouse corneal endothelium (~75% *Neat1* inhibition),^21^ suggesting that the AAV based *Neat1* shRNA is functional.

### 3.2 Neat1 shRNA and factor 8 (F8) gene transfer in Haemophilia A mice

In our next set of experiments, we wished to study the impact of targeted inhibition of *Neat1* by Neat1 shRNA, on mediators of joint damage administered either alone or in combination with liver directed factor 8 gene therapy. We thus packaged the Neat1 shRNA construct into an AAV serotype 5 vector. Previous reports have demonstrated that this serotype is best suited for transgene expression into the articular cartilage.^34^, ^35^ Similarly, a B‐domain deleted human F8 gene (F8‐BDD‐V3, a kind gift from Dr Amit Nathwani, UCL) was packaged into an AAV serotype 8 optimized for high liver directed expression (AAV8K31Q), as described earlier.^22^, ^23^

Groups of haemophilia A mice were pre‐treated with AAV5WT‐U6‐Neat1 shRNA vector (2.5 × 10^11^ vgs/joint, intra‐articular) or AAV8K31Q‐F8‐BDD‐V3 vector (1 × 10^11^ vgs/mouse, intravenous) either alone or in combination, as detailed in Figure 1. A week later, joint injury was performed to mimic hemarthrosis in these animals as described earlier^6^ and the impact on molecular mediators was evaluated after multiple bleeding episodes (Day0, Day14 and Day30).

**FIGURE 2:**
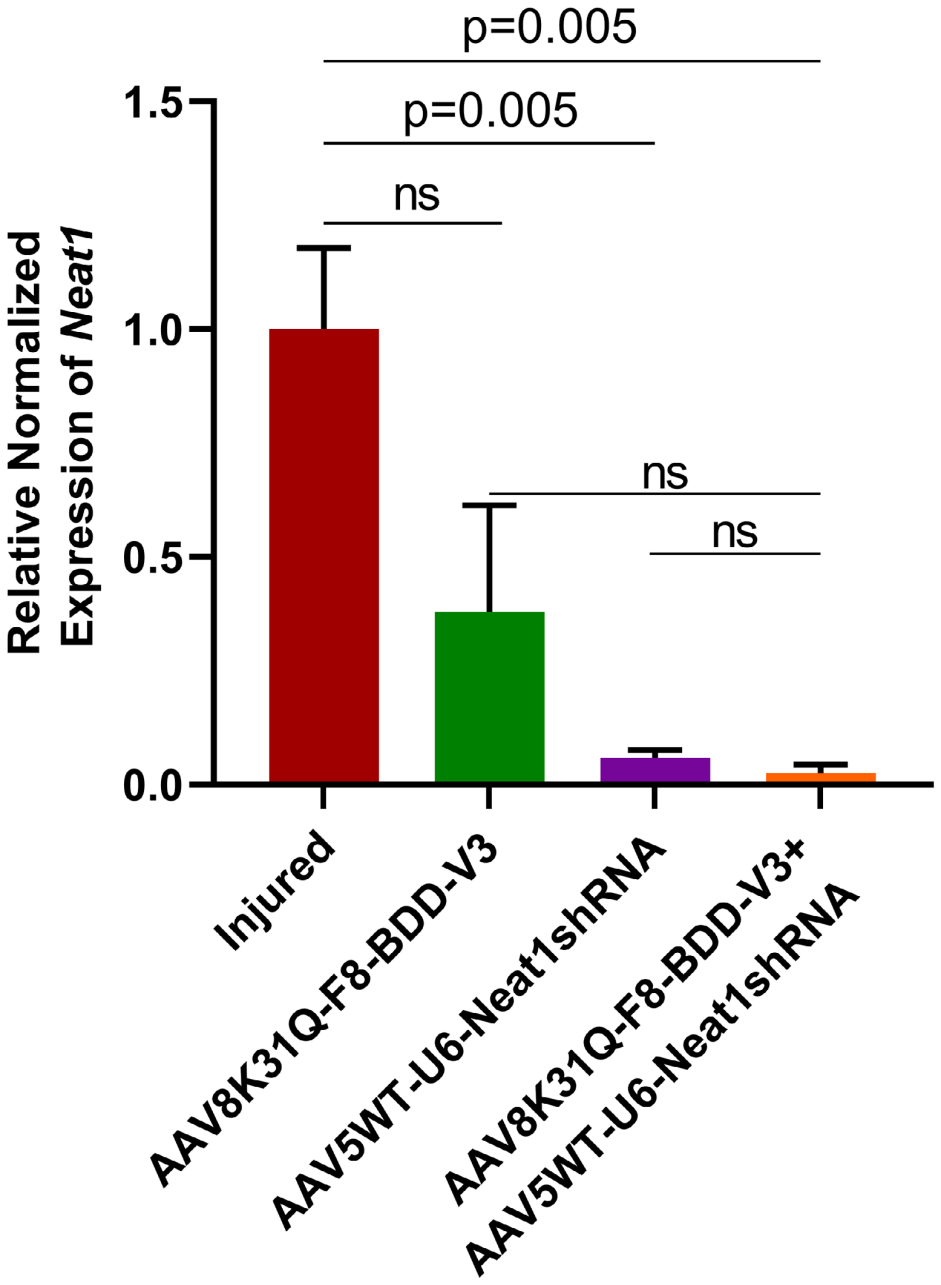
AAV mediated expression of shRNA represses *Neat1* after intra articular gene transfer. Joint tissue was harvested 52 days post vector administration and pooled RNA was isolated from control and injured joints (*n* = 4 mice from each group) using Trizol reagent, respectively. RNA was converted to cDNA for gene expression analysis using quantiTect RT kit. Relative quantification of *Neat1* (*n* = 3 replicates) was performed by quantitative PCR. *Gapdh* levels were used for normalisation of *Neat1* expression. An unpaired Student's *t*‐test was performed for statistical analysis. ns, non significant.

Upon administration with AAV5WT‐U6‐Neat1 shRNA in the articular cartilage, a significant knockdown of *Neat1* was observed in vector‐treated mice in comparison to the mock group (Figure 2). Joint tissues from mice receiving AAV8K31Q‐F8‐BDD‐V3 had slightly reduced expression of *Neat1*, although not significant (*p* = 0.09) compared to the mock group. This could be due to rescue of bleeding phenotype as a result of increased F8 levels^36^ thereby resolving the dysregulation of crucial mediators like *Neat1*. Mice administered with only Neat1 shRNA vector demonstrated a 16 fold downregulation in *Neat1* expression whilst mice receiving combinatorial therapy showed a 40 fold decrease in *Neat1* expression. The combination of F8 reconstitution and shRNA‐mediated *Neat1* knockdown led to a remarkable decrease in *Neat1* levels (~40 fold) in the combination‐treated group when compared to the mock group. This enhanced repression of *Neat1* is possibly due to synergistic effect of FVIII expression and shRNA mediated *Neat1* knockdown in combination treated mice as FVIII reconstitution may directly regulate the molecular mediators and decrease the severity of joint damage.^24^, ^36^ These data further validated the effectiveness of the AAV5 based shRNA vectors to inhibit *Neat1*, in vivo.

**FIGURE 3:**
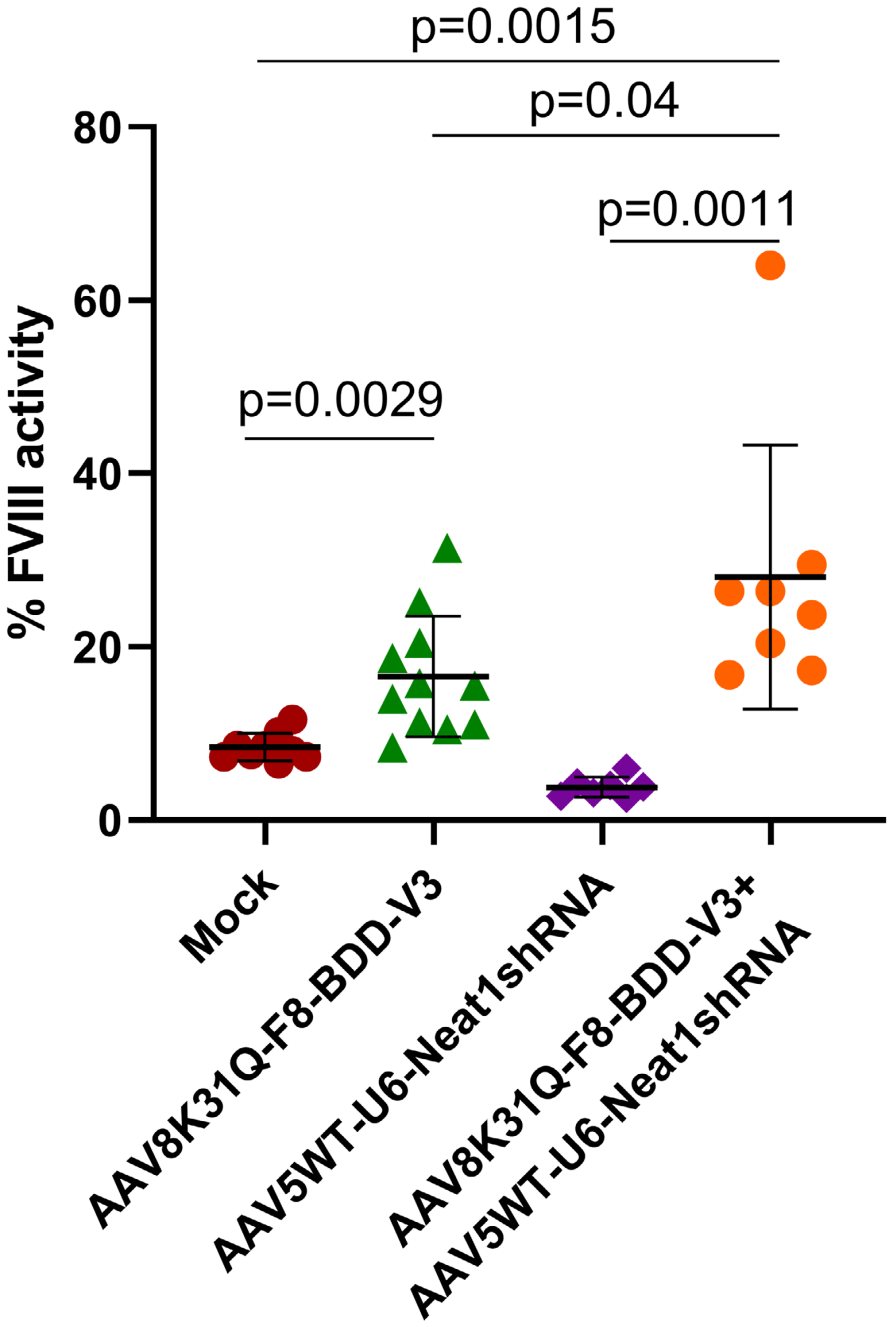
Liver directed F8 gene transfer in haemophilia A mice. Blood samples were collected via retro‐orbital sinus in 3.2% sodium citrate (10:1 vol/vol). Blood samples were then centrifuged at 2000 rpm for 15 min to extract plasma. Factor VIII specific activity in the plasma was measured using clotting based assay (Diagnostica Stago) as per manufacturer's protocol to determine phenotypic rescue in treated mice. FVIII expression in mock (*n* = 9), F8 treated (*n* = 11), Neat1 shRNA treated (*n* = 7), and combinatorial therapy group (*n* = 8) is shown (Mean ± SD). An unpaired Student's *t*‐test was performed for statistical analysis.

We administered only a low dose of F8 vectors (1 × 10^11^ vgs/mice), to study the impact of lncRNA therapy. Our data revealed that the FVIII activity was ~2–3.3 fold higher in mice administered with AAV8K31Q‐F8‐BDD‐V3 vectors (16.5%) either alone or in combination with AAV5WT‐U6‐Neat1 shRNA vectors (28%) when compared to phosphate buffered saline (PBS) injected mice (8.3%) (Figure 3). The FVIII activity level in mock group was observed to be ~8.3% which could be attributed to 10‐fold higher factor V and factor VII levels in mouse plasma compared to human plasma that results in apparent FVIII activity in haemophilia A murine model used in this study. This phenomenon has been observed in previous studies where one stage FVIII clotting assay was used to measure FVIII activity in haemophilia A mice.^7^, ^37^, ^38^, ^39^

### 3.3 Regulation of *Neat1* target proteins in vector treated mice

**FIGURE 4:**
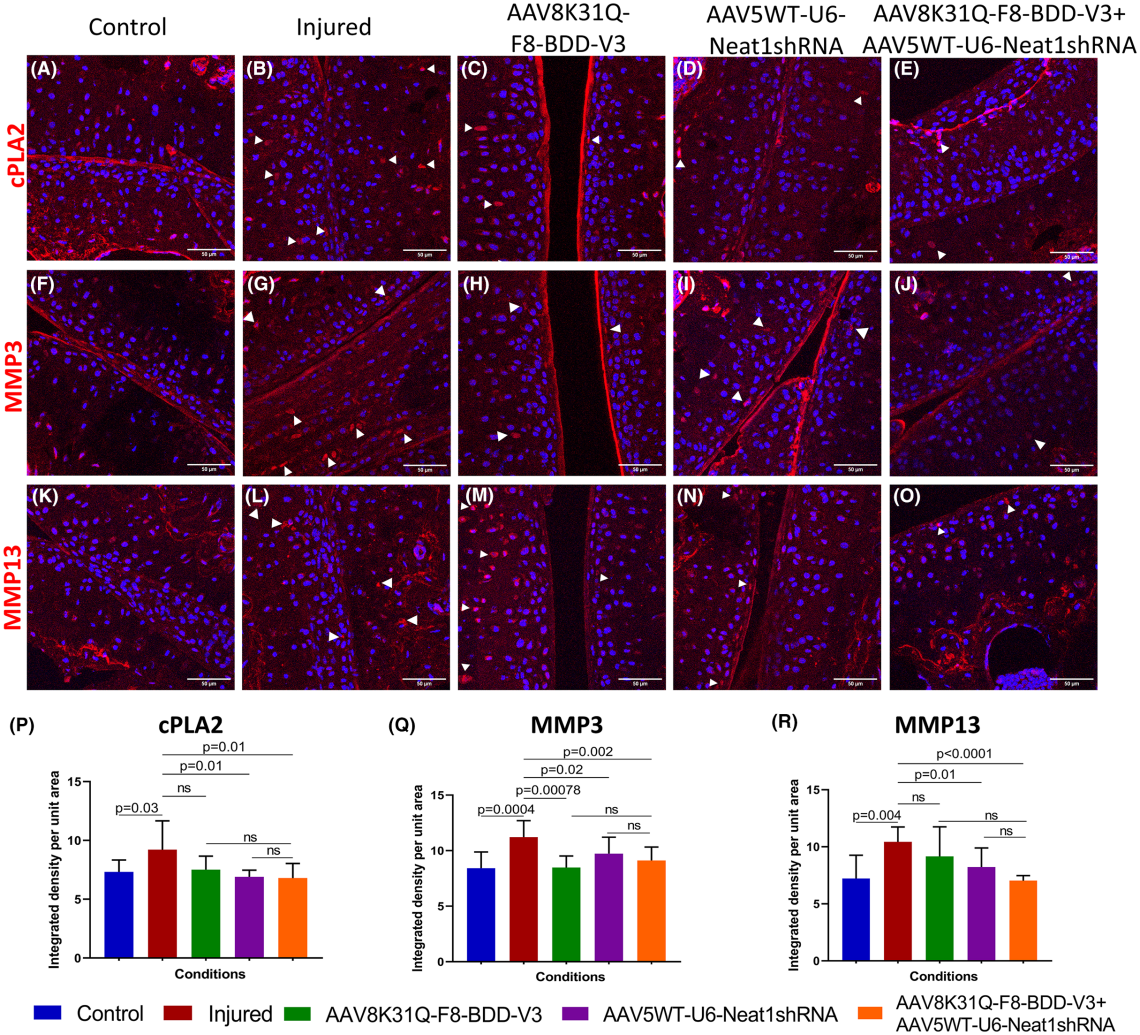
AAV mediated *Neat1* knockdown regulates the target proteins in joint tissue of treated mice. Cryosections (~10 μm) from joint tissue (*n* = 3 animals per group) were obtained (Leica CM1520, Leica Biosystems, Germany) from experimental haemophilia A mice. Primary antibodies against cPLA2 (A–E), MMP 3 (F–J), and MMP13 (K–O) were used to stain these sections. Our results indicate that these molecular regulators are overexpressed in injured joints (denoted by white arrows) and are repressed in Neat1 shRNA treated or combination therapy group. Images were obtained by confocal microscopy (LSM780NLO, Carl Zeiss GmbH). Scale bar is 50 μm. Representative images are shown. Semi‐quantitative analysis of immunofluorescence images for cPLA2 (P), MMP3 (Q), MMP13 (R) (*n* = 3 animals; 3–4 joint sections per group) using Image J software. An unpaired Student's *t*‐test was performed for statistical analysis. ns, non significant.

To further assess the impact of *Neat1* inhibition on molecular mediators of joint disease, we studied the expression of target proteins of *Neat1* such as cPLA2, MMP3 and MMP13^13^, ^40^, ^41^ in the joints by immunohistochemistry. Our data revealed an increased expression of cPLA2 and MMP3 in the injured joints of untreated mice compared to control joint tissue, as demonstrated earlier^8^ (Figure 4A,B,F,G). Interestingly, the mice that received Neat1 shRNA vector alone (Figure 4D,I) or in combination with F8 vector (Figure 4E,J) showed notable reduction in these *Neat1* target proteins (Figure 4) compared to the injured joints of PBS injected mice (Figure 4B,G). However, the expression level of the target proteins cPLA2 and MMP13 were similar in the injured joints of F8 administered mice and mock group (4C, 4 M). The expression of MMP13 was significantly reduced in joint tissue of mice treated with both F8 and Neat1 shRNA vectors (Figure 4O), when compared to untreated injured joints (Figure 4L). Further semi‐quantitative analysis of the immunohistochemical images revealed significant downregulation of the target proteins cPLA2, MMP3 and MMP13 in mice receiving Neat1 shRNA vector and F8 therapy (Figure 4P,Q,R). Upon immunostaining of joint tissue from haemostatically normal mice (C57BL/6J) for cPLA2, MMP3 and MMP13, a similar level of target protein expression was observed in both uninjured and injured joint tissue sections (Figure S6) indicating that repeated injuries in haemostatically normal mice do not lead to arthropathy phenotype as observed earlier.^7^

Upon gross examination, the joint capsules of F8 and Neat1 shRNA vector treated groups demonstrated substantially less bleeding when compared to the untreated injured group (Figure S2). In addition, the mice treated with combination of F8 and shRNA vectors, had reduced synovial hyperplasia and haemorrhage (Figure S3 and S4) and lesser iron deposition (Figure S5).

## 4 DISCUSSION

Although FVIII administration can rescue bleeding phenotype in patients with haemophilia,^42^, ^43^ there have been multiple reports of breakthrough joint bleeds in patients receiving replacement therapy.^44^ This indicates that FVIII expression alone would not be sufficient to prevent development of HA and a thorough understanding of molecular mediators is necessary to understand HA progression. To the best of our knowledge the present study is the first to investigate the effect of lncRNA modulation in the context of molecular mediators of haemophilic joint disease.

LncRNAs, through their interactions with microRNAs, regulate chromatin remodelling and histone modification, and they are involved in a wide variety of biological processes, such as gene transcription, splicing, translation, cell cycle, cell structure maintenance, and apoptosis, among many more.^45^ The dysregulated expression of lncRNA has been linked to several inflammatory diseases.^46^, ^47^ LncRNAs play essential role in the development of bone and cartilage, and the abnormal expression of these lncRNAs in OA cartilage promotes the breakdown of the cartilage extracellular matrix.^48^ Chen et al. revealed that the RA‐associated *Neat1* lncRNA could bind to miR‐204 and increase methylation of the miR‐129 promoter. As a result, miR‐129 is upregulated in rheumatoid arthritis, and this results in a decrease in ERK1/2 phosphorylation and an increase in fibroblast like synoviocytes (FLS) proliferation.^10^ Wang et al. discovered that the expression of *Neat1* and osteopontin (OPN) in osteoarthritis synovial cells were elevated. Following *Neat1* knockout, the expression of MMP13, IL‐6 and IL‐8 in synovial cells decreased, cell proliferation was inhibited, and OPN protein levels decreased.^20^ Previously, our group reported the upregulation of *Neat1* in mouse model of chronic HA and its potential role in the regulation of MMPs.^8^ Since HA is a joint disease that shares phenotypic manifestations noted in OA and RA,^49^ we reasoned that downregulation of *Neat1* may impact joint disease in haemophilia.

*Neat1* knockdown by short interference (si)RNA or shRNA has been applied in multiple studies for knockdown of matrix metalloproteases.^13^, ^20^ For example, Xiao et al. have demonstrated that siRNA mediated knockdown of *Neat1* resulted in decreased expression of MMP3, MMP9 and MMP13 in OA patient derived chondrocytes.^13^ In our work, we utilized AAV5 vectors targeted expression of *Neat1* specific shRNA to regulate the expression of disease modulators, cPLA2 and MMP3 and MMP13. Since, lncRNA based therapy is local and targeted to the joints, we aimed to also reconstitute F8 at low levels and study its impact on markers of cartilage degradation. One of the recent studies has shown that systemic administration of recombinant FVIII (100 IU/kg) in haemophilia A mice significantly reduced the synovitis.^36^ Similarly, the participants from the high dose cohort (6 × 10^13^ vgs/kg) in the AAV5‐hFVIII‐SQ clinical trial showed complete resolution of bleeding in the target joints 48 months after vector administration.^50^

The immunostaining data for the *Neat1* target proteins demonstrated significant attenuation of the inflammatory marker cPLA2 (*p* = 0.01), and cartilage degenerative enzymes MMP3 (*p* = 0.002) and MMP13 (*p* < 0.0001) in combinatorial gene transfer group compared to untreated injured joints. This demonstrates the synergistic effect of F8 gene augmentation and *Neat1* inhibition by possibly strengthening the baseline coagulation function and anti‐inflammatory/ chondroprotective function in mitigating the arthropathic phenotype. The use of AAV vectors for combinatorial gene transfer and targeting multiple disease pathways has been demonstrated in other diseases like alpha‐1 antitrypsin (AAT) deficiency, retinitis pigmentosa and various age related diseases.^51^, ^52^, ^53^ Nonetheless, such strategies require careful consideration of the choice of AAV serotype based on the target tissue,^54^ immune response to the AAV vectors and their dosage optimisation.^55^, ^56^

Our study has certain limitations. We injected animals with AAV F8/Neat1 shRNA vectors prior to initiation of joint injury and documented its effect on molecular mediators. Whilst this is advantageous to directly measure the impact of F8 gene augmentation and *Neat1* inhibition, a further validation with on‐demand administration of vectors, at different stages of arthropathy development may be required to comprehensively dissect the impact of this intervention at the molecular level in the joints. Since many of these lncRNAs and associated pathways are conserved, this knowledge can benefit a variety of other inflammatory joint diseases such as OA and Juvenile Idiopathic Arthritis.

## AUTHOR CONTRIBUTIONS

**Pratiksha Sarangi:** Formal analysis (lead); investigation (lead); methodology (lead). **Mohankumar B. Senthilkumar:** Formal analysis (supporting); investigation (supporting); writing – original draft (supporting). **Sonal Amit:** Formal analysis (supporting); investigation (supporting); methodology (supporting). **Narendra Kumar:** Investigation (supporting); supervision (supporting). **Giridhara R. Jayandharan:** Conceptualization (lead); funding acquisition (lead); project administration (lead); resources (lead); writing – original draft (equal); writing – review and editing (lead).

## FUNDING INFORMATION

This work was supported by a research grant from Science and Engineering Research Board (SERB) “CRG/2019/001211”.

## CONFLICT OF INTEREST STATEMENT

PS, MK, NK, GRJ have applied for patents on AAV technology for gene therapy and few technologies have been licensed.

## Supporting information


Data S1.

## Data Availability

All relevant data supporting the findings of this study are presented in the article and its supplementary information. Raw data is available from the corresponding author (GRJ) upon reasonable request.

## References

[1] Castaman G , Matino D . Hemophilia A and B: molecular and clinical similarities and differences. Haematologica. 2019;104(9):1702‐1709. doi:10.3324/haematol.2019.221093 31399527 PMC6717582

[2] Gualtierotti R , Solimeno LP , Peyvandi F . Hemophilic arthropathy: current knowledge and future perspectives. J Thromb Haemost. 2021;19(9):2112‐2121. doi:10.1111/jth.15444 34197690 PMC8456897

[3] Zhou JY , Barnes RF , Foster G , Iorio A , Cramer TJ , von Drygalski A . Joint bleeding tendencies in adult patients with hemophilia: it's not all pharmacokinetics. Clin Appl Thromb Hemost. 2019;25:1076029619862052. doi:10.1177/1076029619862052 31298044 PMC6714908

[4] Oldenburg J . Optimal treatment strategies for hemophilia: achievements and limitations of current prophylactic regimens. Blood. 2015;125(13):2038‐2044. doi:10.1182/blood-2015-01-528414 25712992

[5] Blobel CP , Haxaire C , Kalliolias GD , DiCarlo E , Salmon J , Srivastava A . Blood‐induced arthropathy in hemophilia: mechanisms and heterogeneity. Semin Thromb Hemost. 2015;41(8):832‐837. doi:10.1055/s-0035-1564445 26451745

[6] Sen D , Chapla A , Walter N , Daniel V , Srivastava A , Jayandharan GR . Nuclear factor (NF)‐κB and its associated pathways are major molecular regulators of blood‐induced joint damage in a murine model of hemophilia. J Thromb Haemost. 2013;11(2):293‐306. doi:10.1111/jth.12101 23231432

[7] Senthilkumar MB , Sarangi P , Amit S , Senguttuvan S , Kumar N , Jayandharan GR . Targeted delivery of miR125a‐5p and human factor VIII attenuates molecular mediators of hemophilic arthropathy. Thromb Res. 2023;231:8‐16. doi:10.1016/j.thromres.2023.09.008 37741049

[8] Sarangi P , Senthilkumar MB , Kumar N , Senguttuvan S , Vasudevan M , Jayandharan GR . Potential role of long non‐coding RNA H19 and Neat1 in haemophilic arthropathy. J Cell Mol Med. 2023;7(12):1745‐1749. doi:10.1111/jcmm.17770 PMC10273061 37183540

[9] Liwu ZHOU , Yang WAN , Cheng Q , Ben SHI , Zhang L , Shuo CHEN . The expression and diagnostic value of LncRNA H19 in the blood of patients with osteoarthritis. Iran J Public Health. 2020;49(8):1494‐1501. doi:10.18502/ijph.v49i8.3893 33083326 PMC7554376

[10] Xiao J , Wang R , Zhou W , Cai X , Ye Z . LncRNA NEAT1 regulates the proliferation and production of the inflammatory cytokines in rheumatoid arthritis fibroblast‐like synoviocytes by targeting miR‐204‐5p. Hum Cell. 2021;34(2):372‐382. doi:10.1007/s13577-020-00461-4 33394349

[11] Xie F , Liu YL , Chen XY , et al. Role of MicroRNA, LncRNA, and exosomes in the progression of osteoarthritis: a review of recent literature. Orthop Surg. 2020;12(3):708‐716. doi:10.1111/os.12690 32436304 PMC7307224

[12] Yang B , Xu L , Wang S . Regulation of lncRNA‐H19/miR‐140‐5p in cartilage matrix degradation and calcification in osteoarthritis. Ann Palliat Med. 2020;9(4):1896‐1904. doi:10.21037/apm-20-929 32576007

[13] Xiao P , Zhu XU , Sun J , et al. LncRNA NEAT1 regulates chondrocyte proliferation and apoptosis via targeting miR‐543/PLA2G4A axis. Hum Cell. 2021;34(1):60‐75. doi:10.1007/s13577-020-00433-8 33040229

[14] Mahmoudi Z , Karamali N , Roghani SA , et al. Efficacy of DMARDs and methylprednisolone treatment on the gene expression levels of HSPA5, MMD, and non‐coding RNAs MALAT1, H19, miR‐199a‐5p, and miR‐1‐3p, in patients with rheumatoid arthritis. Int Immunopharmacol. 2022;108:108878. doi:10.1016/j.intimp.2022.108878 35623291

[15] Chatterjee S , Bhattcharjee D , Misra S , Saha A , Bhattacharyya NP , Ghosh A . Increase in MEG3, MALAT1, NEAT1 significantly predicts the clinical parameters in patients with rheumatoid arthritis. Perinat Med. 2020;17(6):445‐457. doi:10.2217/pme-2020-0009 33026292

[16] Fu X , Song G , Ni R , et al. LncRNA‐H19 silencing suppresses synoviocytes proliferation and attenuates collagen‐induced arthritis progression by modulating miR‐124a. Rheumatology. 2021;60(1):430‐440. doi:10.1093/rheumatology/keaa395 32810279

[17] An H , Williams NG , Shelkovnikova TA . NEAT1 and paraspeckles in neurodegenerative diseases: a missing lnc found? Non‐coding RNA Res. 2018;3(4):243‐252. doi:10.1016/j.ncrna.2018.11.003 PMC6257911 30533572

[18] Guo T , Xing Y , Chen Z . Long non‐coding RNA NEAT1 knockdown alleviates rheumatoid arthritis by reducing IL‐18 through p300/CBP repression. Inflammation. 2022;45(1):100‐115. doi:10.1007/s10753-021-01531-x 34773548

[19] Zhi LQ , Zhong Q , Ma JB , Xiao L , Yao SX , Wang X . LncRNA H19 inhibitor represses synovial cell proliferation and apoptosis in rats with rheumatoid arthritis via notch signaling pathway. Eur Rev Med Pharmacol Sci. 2020;24(15):7921. doi:10.26355/eurrev_202008_22456 32767314

[20] Wang Q , Wang W , Zhang F , Deng Y , Long Z . NEAT1/miR‐181c regulates osteopontin (OPN)‐mediated synoviocyte proliferation in osteoarthritis. J Cell Biochem. 2017;118(11):3775‐3784. doi:10.1002/jcb.26025 28379604

[21] Wang Q , Dou S , Zhang B , et al. Heterogeneity of human corneal endothelium implicates lncRNA NEAT1 in Fuchs endothelial corneal dystrophy. Mol Ther Nucleic Acids. 2022;27:880‐893. doi:10.1016/j.omtn.2022.01.005 35141048 PMC8807987

[22] Maurya S , Mary B , Jayandharan GR . Rational engineering and preclinical evaluation of neddylation and SUMOylation site modified adeno‐associated virus vectors in murine models of hemophilia B and leber congenital amaurosis. Hum Gene Ther. 2019;30(12):1461‐1476. doi:10.1089/hum.2019.164 31642343 PMC6919284

[23] Mary B , Maurya S , Kumar M , Bammidi S , Kumar V , Jayandharan GR . Molecular engineering of adeno‐associated virus capsid improves its therapeutic gene transfer in murine models of hemophilia and retinal degeneration. Mol Pharm. 2019;16(11):4738‐4750. doi:10.1021/acs.molpharmaceut.9b00959 31596095 PMC7035104

[24] Sen D , Jayandharan GR . MicroRNA‐15b modulates molecular mediators of blood induced arthropathy in hemophilia mice. Int J Mol Sci. 2016;17(4):492. doi:10.3390/ijms17040492 27070581 PMC4848948

[25] Hakobyan N , Enockson C , Cole AA , Sumner DR , Valentino LA . Experimental haemophilic arthropathy in a mouse model of a massive haemarthrosis: gross, radiological and histological changes. Haemophilia. 2008;14(4):804‐809. doi:10.1111/j.1365-2516.2008.01689.x 18422608

[26] Cleuren AC , van der Linden IK , de Visser YP , Wagenaar GT , Reitsma PH , van Vlijmen BJ . 17α‐Ethinylestradiol rapidly alters transcript levels of murine coagulation genes via estrogen receptor α. J Thromb Haemost. 2010;8(8):1838‐1846. doi:10.1111/j.1538-7836.2010.03930.x 20524981

[27] Valentino LA , Hakobyan N . Histological changes in murine haemophilic synovitis: a quantitative grading system to assess blood‐induced synovitis. Haemophilia. 2006;12(6):654‐662. doi:10.1111/j.1365-2516.2006.01348.x 17083517

[28] Vøls KK , Kjelgaard‐Hansen M , Ley CD , Hansen AK , Petersen M . Bleed volume of experimental knee haemarthrosis correlates with the subsequent degree of haemophilic arthropathy. Haemophilia. 2019;25(2):324‐333. doi:10.1111/hae.13672 30648774

[29] Inagawa K , Oohashi T , Nishida K , et al. Optical imaging of mouse articular cartilage using the glycosaminoglycans binding property of fluorescent‐labeled octaarginine. Osteoarthr Cartil. 2009;17(9):1209‐1218. doi:10.1016/j.joca.2009.03.010 19332175

[30] Wang W , Guo ZH . Downregulation of lncRNA NEAT1 ameliorates LPS‐induced inflammatory responses by promoting macrophage M2 polarization via miR‐125a‐5p/TRAF6/TAK1 axis. Inflammation. 2020;43:1548‐1560. doi:10.1007/s10753-020-01231-y 32388658

[31] Ma F , Lei YY , Ding MG , Luo LH , Xie YC , Liu XL . LncRNA NEAT1 interacted with DNMT1 to regulate malignant phenotype of cancer cell and cytotoxic T cell infiltration via epigenetic inhibition of p53, cGAS, and STING in lung cancer. Front Genet. 2020;11:250. doi:10.3389/fgene.2020.00250 32296457 PMC7136539

[32] Taxman DJ , Moore CB , Guthrie EH , Huang MT . Short hairpin RNA (shRNA): design, delivery, and assessment of gene knockdown. RNA therapeutics. Methods Mol Biol. 2010;629:139‐156. doi:10.1007/978-1-60761-657-3_10 PMC3679364 20387148

[33] Qing K , Mah C , Hansen J , Zhou S , Dwarki V , Srivastava A . Human fibroblast growth factor receptor 1 is a co‐receptor for infection by adeno‐associated virus 2. Nat Med. 1999;5(1):71‐77. doi:10.1038/4758 9883842

[34] Khoury M , Adriaansen J , Vervoordeldonk MJBM , et al. Inflammation‐inducible anti‐TNF gene expression mediated by intra‐articular injection of serotype 5 adeno‐associated virus reduces arthritis. J Gene Med. 2007;9(7):596‐604. doi:10.1002/jgm.1053 17514770

[35] Wu X , Lai Y , Chen S , et al. Kindlin‐2 preserves integrity of the articular cartilage to protect against osteoarthritis. Nat Aging. 2022;2(4):332‐347. doi:10.1038/s43587-021-00165-w 37117739

[36] Zhang F , Yan X , Li M , et al. Exploring the potential feasibility of intra‐articular adeno‐associated virus‐mediated gene therapy for hemophilia arthropathy. Hum Gene Ther. 2020;31(7–8):448‐458. doi:10.1089/hum.2019.355 32079420

[37] Doering CB , Parker ET , Healey JF , Craddock HN , Barrow RT , Lollar P . Expression and characterization of recombinant murine factor VIII. J Thomb Haemost. 2002;88(9):450‐458. doi:10.1055/s-0037-1613237 12353075

[38] Chao BN , Baldwin WH , Healey JF , et al. Characterization of a genetically engineered mouse model of hemophilia a with complete deletion of the F8 gene. J Thomb Haemost. 2016;14(2):346‐355. doi:10.1111/jth.13202 PMC4755856 26588198

[39] Batsuli G , Ito J , Mercer R , et al. Anti‐C1 domain antibodies that accelerate factor VIII clearance contribute to antibody pathogenicity in a murine hemophilia a model. J Thomb Haemost. 2018;16(9):1779‐1788. doi:10.1111/jth.14233 PMC6123829 29981270

[40] Sommerfelt RM , Feuerherm AJ , Jones K , Johansen B . Cytosolic phospholipase A2 regulates TNF‐induced production of joint destructive effectors in synoviocytes. PLoS One. 2013;8(12):e83555. doi:10.1371/journal.pone.0083555 24349530 PMC3861525

[41] Tai N , Kuwabara K , Kobayashi M , et al. Cytosolic phospholipase A2 alpha inhibitor, pyrroxyphene, displays anti‐arthritic and anti‐bone destructive action in a murine arthritis model. Inflamm Res. 2010;59(1):53‐62. doi:10.1007/s00011-009-0069-8 19655230

[42] Gringeri A , Lundin B , Von Mackensen S , Mantovani L , Mannucci PM . Primary and secondary prophylaxis in children with haemophilia a reduces bleeding frequency and arthropathy development compared to on‐demand treatment: a 10‐year, randomized, clinical trial. J Thromb Haemost. 2009;7:114‐115. doi:10.1111/j.538-7836.2009.03473_1.x

[43] Manco‐Johnson MJ , Abshire TC , Shapiro AD , et al. Prophylaxis versus episodic treatment to prevent joint disease in boys with severe hemophilia. N Engl J Med. 2007;357(6):535‐544. doi:10.1056/NEJMoa067659 17687129

[44] van Vulpen LF , Thomas S , Keny SA , Mohanty SS . Synovitis and synovectomy in haemophilia. Haemophilia Suppl. 2021;3:96‐102. doi:10.1111/hae.14025 PMC7984224 32490595

[45] Rinn JL , Chang HY . Genome regulation by long noncoding RNAs. Annu Rev Biochem. 2012;81:145‐166. doi:10.1146/annurev-biochem-051410-092902 22663078 PMC3858397

[46] Nandwani A , Rathore S , Datta M . LncRNAs in cancer: regulatory and therapeutic implications. Cancer Lett. 2021;501:162‐171. doi:10.1016/j.canlet.2020.11.048 33359709

[47] Jiang MC , Ni JJ , Cui WY , Wang BY , Zhuo W . Emerging roles of lncRNA in cancer and therapeutic opportunities. Am J Cancer Res. 2019;9(7):1354‐1366. 31392074 PMC6682721

[48] Xing D , Liang JQ , Li Y , et al. Identification of long noncoding RNA associated with osteoarthritis in humans. Orthop Surg. 2014;6(4):288‐293. doi:10.1111/os.12147 25430712 PMC6583210

[49] Toenges R , Wittenbrink A , Miesbach W . Biomarkers and immunological parameters in haemophilia and rheumatoid arthritis patients: a comparative multiplexing laboratory study. Haemophilia. 2021;27(1):e119‐e126. doi:10.1111/hae.14200 33210410

[50] Pasi KJ , Rangarajan S , Mitchell N , et al. Multiyear follow‐up of AAV5‐hFVIII‐SQ gene therapy for hemophilia a. N Engl J Med. 2020;382(1):29‐40. doi:10.1056/NEJMoa1908490 31893514

[51] Li C , Xiao P , Gray SJ , Weinberg MS , Samulski RJ . Combination therapy utilizing shRNA knockdown and an optimized resistant transgene for rescue of diseases caused by misfolded proteins. Proc Natl Acad Sci. 2011;108(34):14258‐14263. doi:10.1073/pnas.1109522108 21844342 PMC3161599

[52] Fortuny C , Byrne L , Dalkara D , Lee T , Ozturk BE . AAV‐mediated combination therapy of neurotrophic and anti‐apoptotic factors in a mouse model of inherited retinal degeneration. Invest Ophthalmol Vis Sci. 2013;54(15):2746.23532527

[53] Davidsohn N , Pezone M , Vernet A , et al. A single combination gene therapy treats multiple age‐related diseases. Proc Natl Acad Sci. 2019;116(47):23505‐23511. doi:10.1073/pnas.1910073116 31685628 PMC6876218

[54] Naso MF , Tomkowicz B , Perry WL III , Strohl WR . Adeno‐associated virus (AAV) as a vector for gene therapy. BioDrugs. 2017;31(4):317‐334. doi:10.1007/s40259-017-0234-5 28669112 PMC5548848

[55] Colella P , Ronzitti G , Mingozzi F . Emerging issues in AAV‐mediated in vivo gene therapy. Mol Ther Methods Clin Dev. 2018;8:87‐104. doi:10.1016/j.omtm.2017.11.007 29326962 PMC5758940

[56] Hareendran S , Balakrishnan B , Sen D , Kumar S , Srivastava A , Jayandharan GR . Adeno‐associated virus (AAV) vectors in gene therapy: immune challenges and strategies to circumvent them. Rev Med Virol. 2013;23(6):399‐413. doi:10.1002/rmv.1762 24023004

