# Supplementary material for: AAV mediated repression of Neat1 lncRNA combined with F8 gene augmentation mitigates pathological mediators of joint disease in haemophilia
Source: J Cell Mol Med. 2024 Jun 12;28(11):e18460. doi: 10.1111/jcmm.18460 (PMC11167708; doi:10.1111/jcmm.18460)
Supplement: Supplementary file 1 — Data S1. [file JCMM-28-e18460-s001.docx]

**Supplementary Methods:**

I**mmunohistochemistry for *Neat1* target proteins in the joint tissue of hemostatically normal mice:**

C57BL/6J mice were procured from Jackson Laboratory (Maine, USA) and housed in animal facility with food and water. The hemostatically normal mice (strain C57BL/6J) (8-12 weeks) were utilized for this study. The levels of *Neat1* target proteins cPLA2, MMP3 and MMP13 in the injured joints of hemostatically normal mice were evaluated to assess their expression in response to needle injury. The right knee joint was injured using 31G needle as described previously^1^ on Day0, Day14, and Day30 whereas contralateral left knee joint was used as uninjured control. On Day45, the joint tissues were harvested, and processed further for immunostaining of target proteins as highlighted in the methods section. The immune-fluorescence images for the joint tissue probed with cPLA2, MMP3 and MMP13 is shown in Supplementary Figure S6.

**Supplementary Figure:**

**
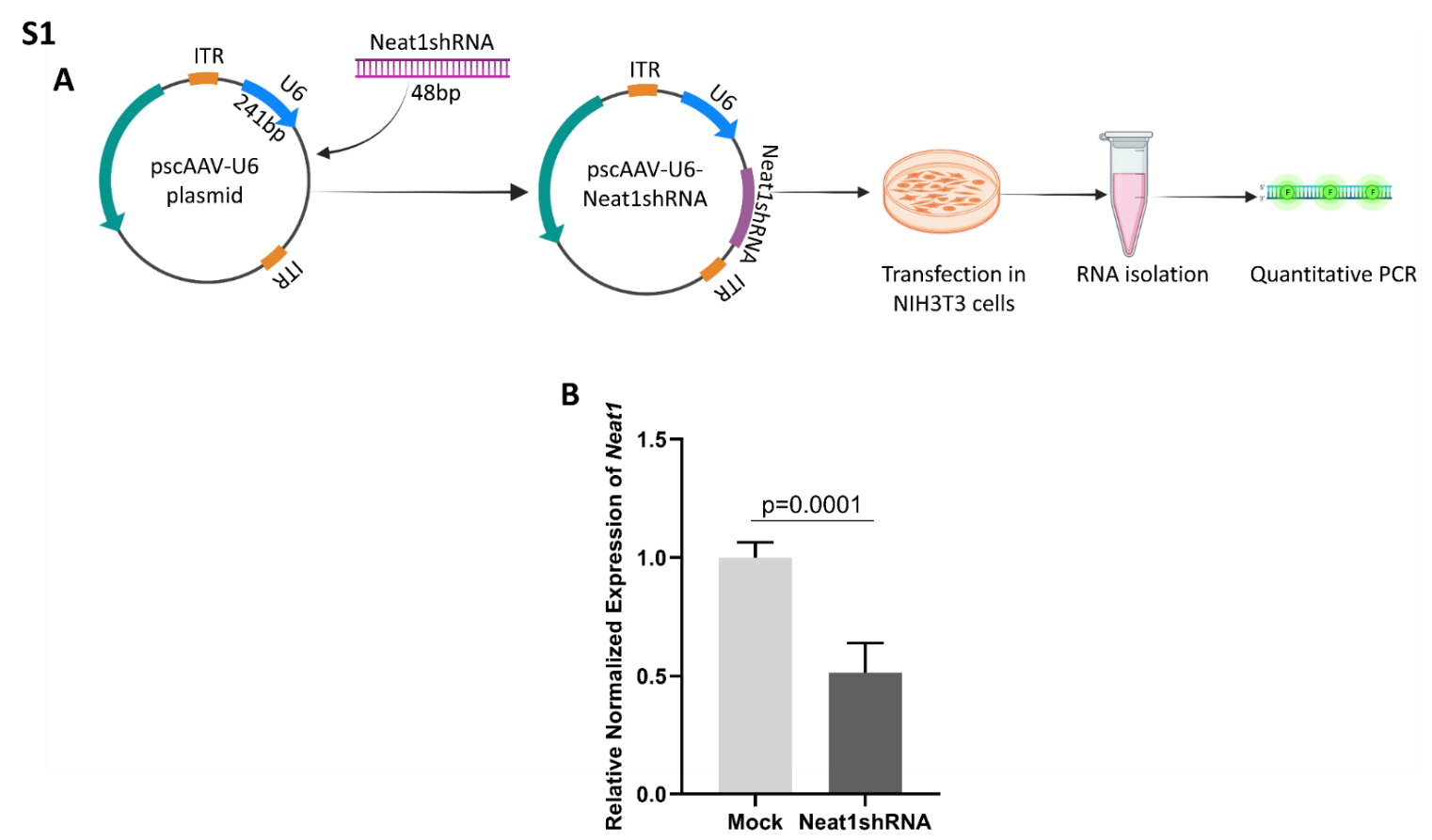
**

**Figure S1. Design of an AAV based shRNA vector to *Neat1* and its validation *in vitro***

The Neat1 shRNA sequence [48bp] was sub-cloned to the AAV backbone [694bp] under the control of a U6 promoter [241bp] (A). The expression of Neat1 shRNA was confirmed by transfection of the cloned plasmid in NIH3T3 cells. Approximately 500ng amount of plasmid was transfected into NIH3T3 cells using a Fugene^HD^ transfection reagent. 48 hrs later we isolated the RNA using Trizol method and cDNA was synthesized from total RNA using quantitect reverse transcription (RT) kit (Qiagen). A relative quantification of *Neat1* (n=9 replicates) was performed using quantitative PCR [CFX96, Bio-Rad Laboratories, California, USA]. *Gapdh* levels were measured and further used to normalize the expression analysis (B). Representative data for relative normalized expression of *Neat1* is shown. Data represents mean±SD from 3 replicate analysis.

**
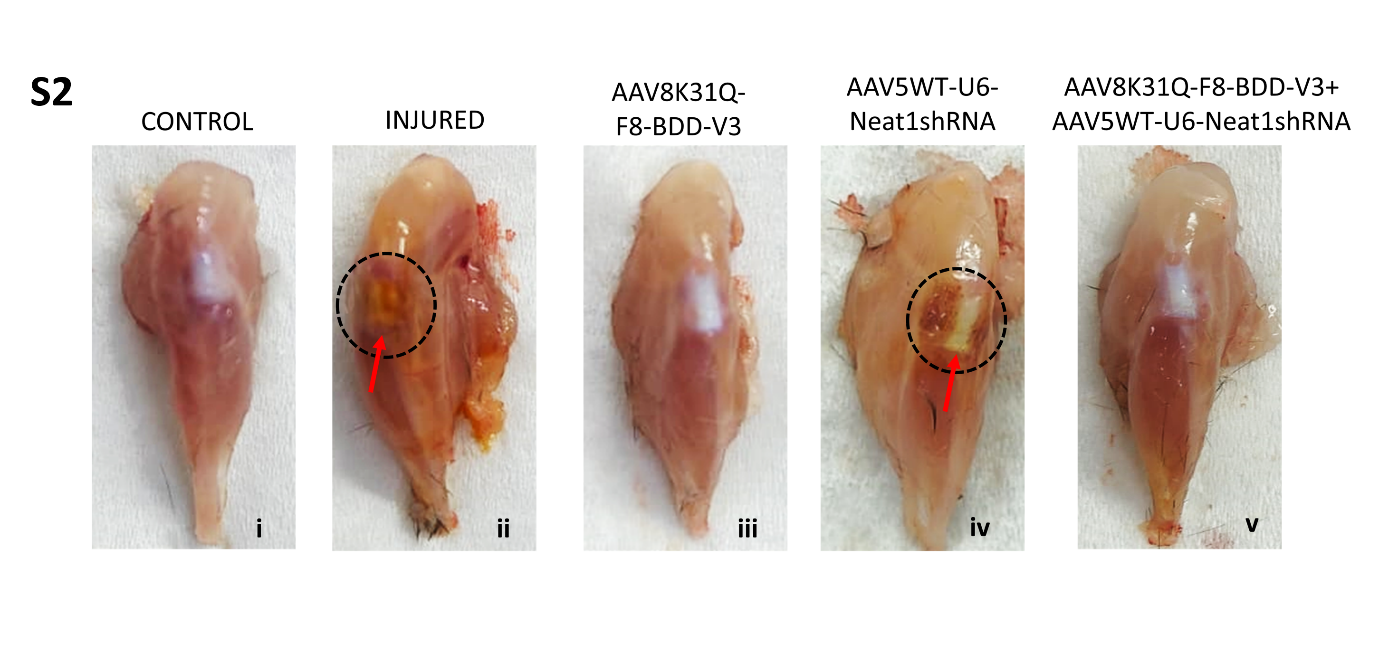
**

**Figure S2. Morphological and histological assessment of target joints that received F8 or Neat1 shRNA vectors.** Gross examination of joint capsules from control (i), injured (ii), F8 treated (iii), Neat1 shRNA injected (iv), and combinatorial vector (v), groups. Red arrows indicate discolouration of the cartilage.

**
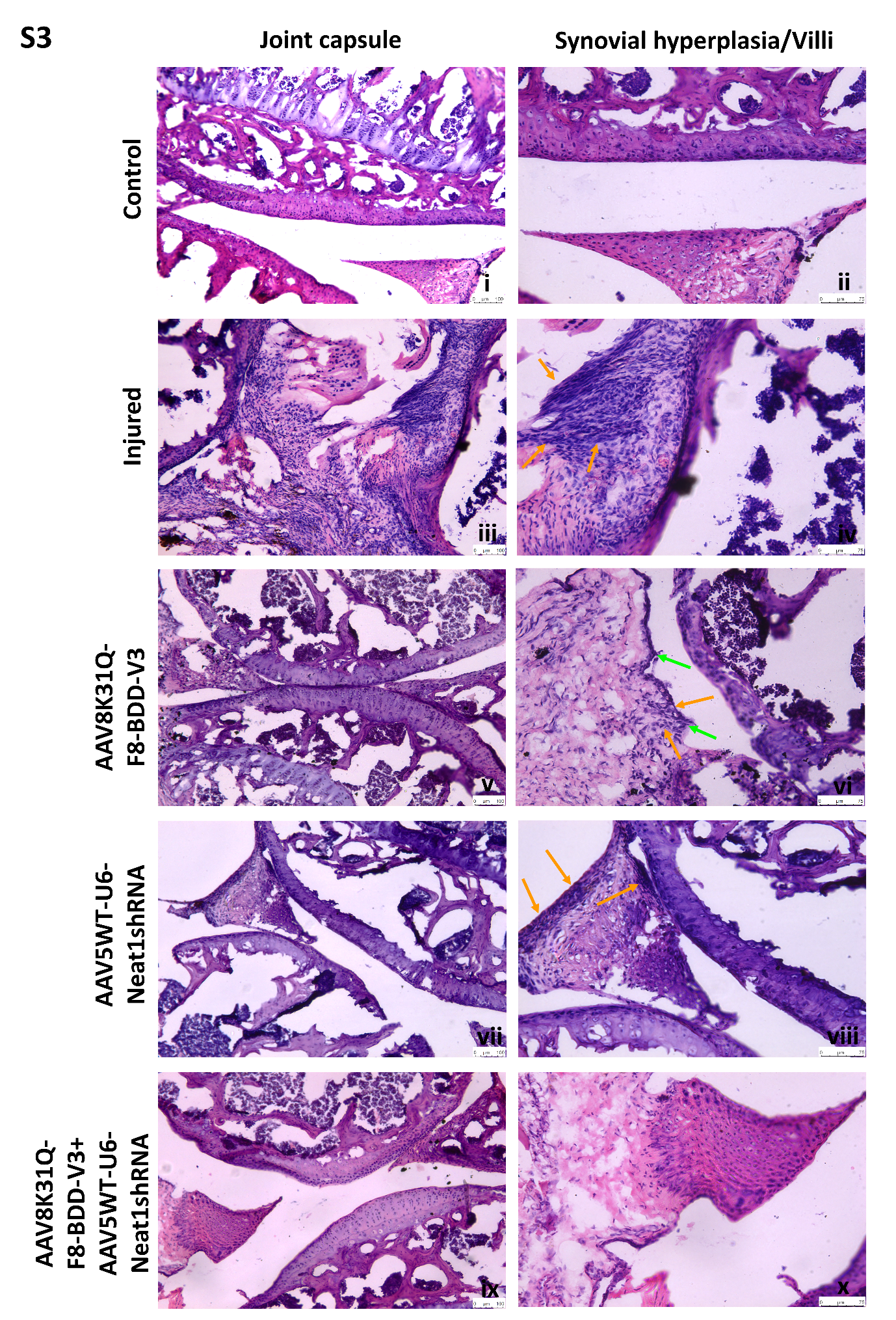
**

**Figure S3. Joint architecture of knee joint in mice treated with AAV based Neat1 shRNA or F8 gene augmentation.** Joint tissue sections (~10µm) were stained with H&E for histological analysis. Representative images of various experimental groups are shown. Magnified images of joint capsule of different experimental groups (i, iii, v, vii, ix) are depicted in the right panel for synovial hyperplasia/villi (ii, iv, vi, viii, x). Orange arrows indicate synovial hyperplasia and green arrows show villus. Scale bar is 100 µm for 100X magnification and 75 µm for 200X magnification. Images were obtained using inverted light microscope (DMi8, Leica Microsystems, Wetzlar, Germany).

**
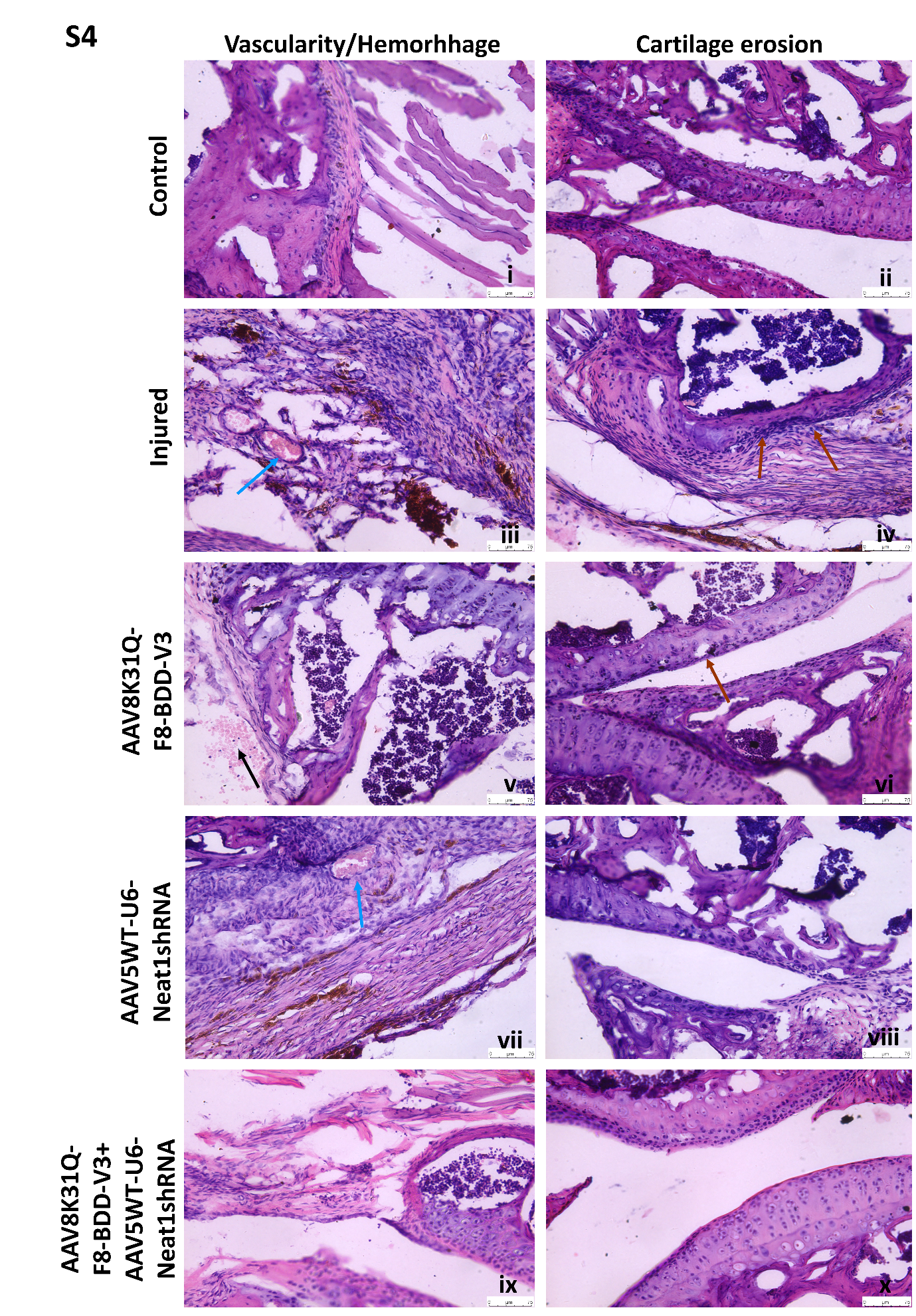
**

**Figure S4. Angiogenesis and cartilage erosion in knee joint of mice treated with AAV based Neat1 shRNA or F8 gene augmentation.** Joint tissue sections (~10µm) were stained with H&E for histological analysis. Representative images of various experimental groups are shown. Blue arrows refer to increased vasculature, black arrow refers to hemorrhage and brown arrows indicate cartilage erosion. Scale bar is 75 µm. Images were obtained using inverted light microscope (DMi8, Leica Microsystems, Wetzlar, Germany).

**
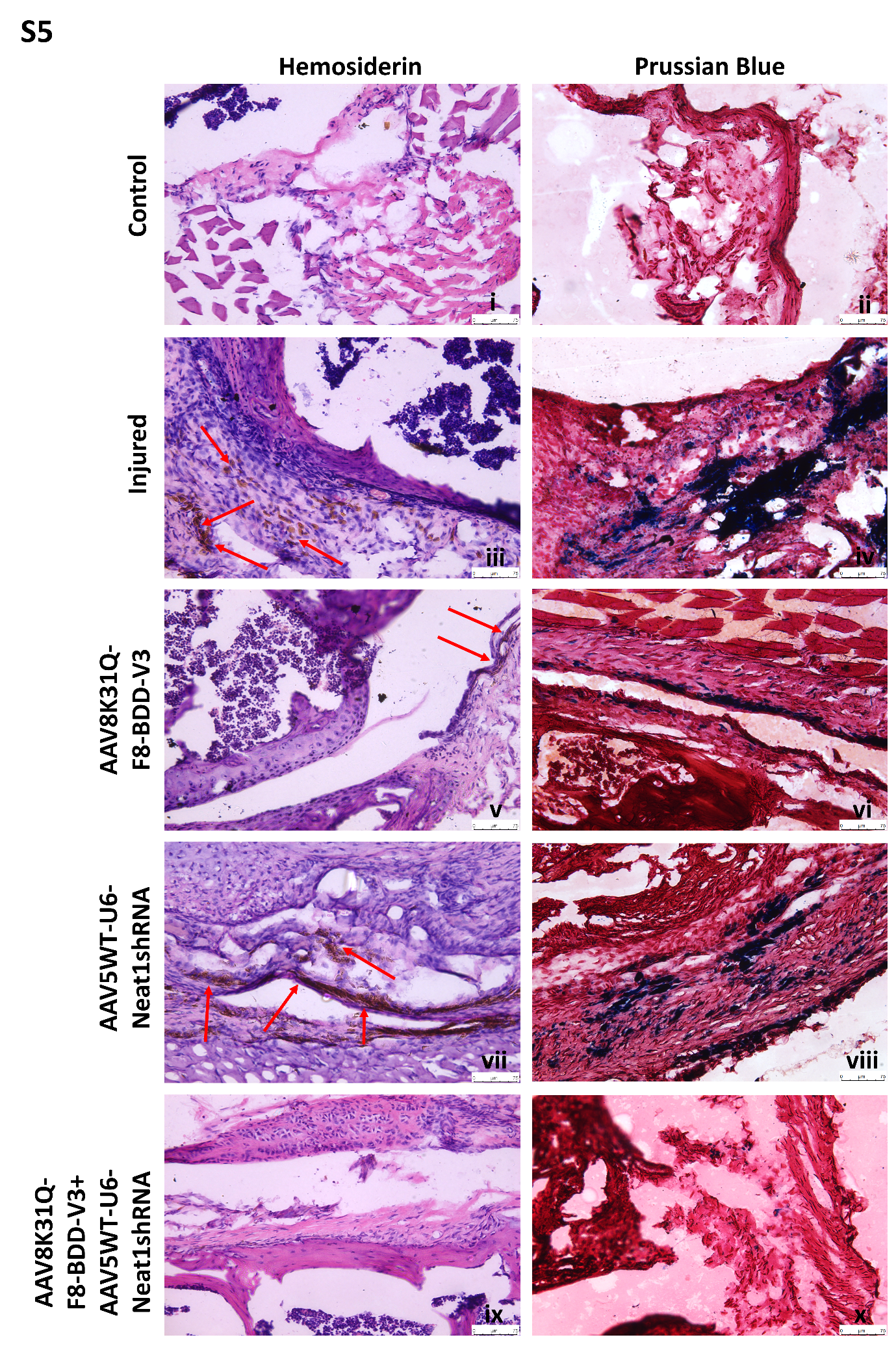
**

**Figure S5. Iron deposition in knee joint of mice treated with AAV based Neat1 shRNA or F8 gene augmentation.** Joint tissue sections (~10µm) were stained with H&E and Prussian blue for histological analysis. Representative images of various experimental groups are shown. Red arrows show hemosiderin deposition. Scale bar is 75 µm. Images were obtained using inverted light microscope (DMi8, Leica Microsystems, Wetzlar, Germany).


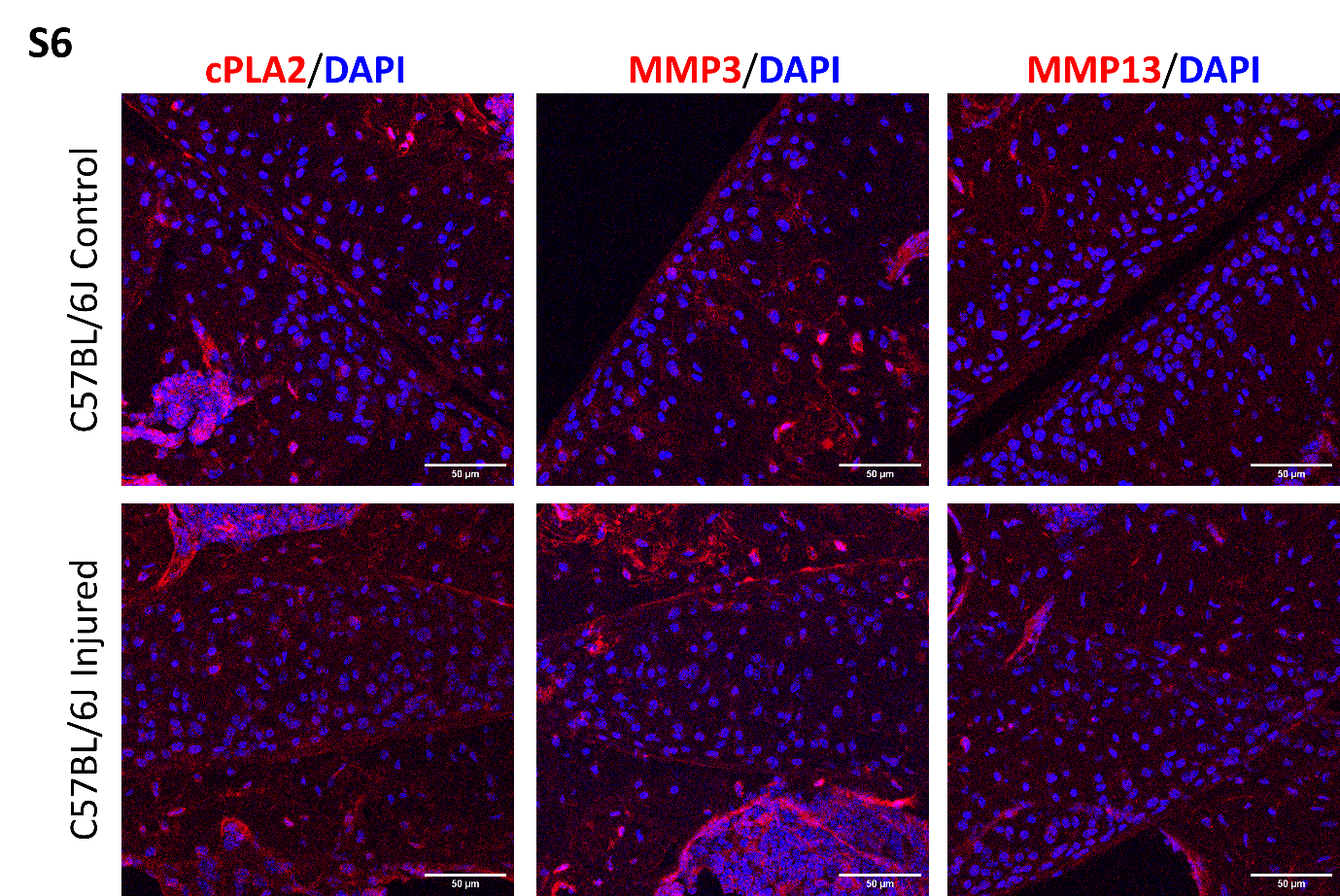


**Figure S6. *Neat1* lncRNA target proteins demonstrate similar levels of expression in the injured and control joint tissues of C57BL6J mice.** Control and injured joint tissues were harvested and decalcified in 14% etheylenediaminetetraacetic acid (EDTA) for 10 days. Decalcified joint tissues were used for obtaining cryosections (~10µm thickness) which were probed with cPLA2, MMP3 and MMP13 antibodies for immunostaining as described in the methods section. Immunostaining showed similar expression level in both the control and injured joints of C57BL6J mice for cPLA2, MMP3 and MMP13 as observed in control joints of hemophilia A mice. Injured joint from hemophilia A mice served as positive control (Figure 4).The fluorescence imaging was performed using confocal imaging system (Zeiss LSM780, Carl Zeiss AG, Germany). Scale bar is 50µm.

**References:**

1. Sen D, Jayandharan GR. MicroRNA-15b modulates molecular mediators of blood induced arthropathy in hemophilia mice. *Int J Mol Sci.* 2016;17, 492. doi: [10.3390/ijms17040492](https://doi.org/10.3390/ijms17040492)
